# “Locked” cancer cells are more sensitive to chemotherapy

**DOI:** 10.1002/btm2.10130

**Published:** 2019-06-10

**Authors:** Yaqi Lyu, Qingqing Xiao, Yi Li, Yubing Wu, Wei He, Lifang Yin

**Affiliations:** ^1^ Department of Pharmaceutics School of Pharmacy, China Pharmaceutical University Nanjing China

**Keywords:** dual nanomedicines, extracellular matrix, matrix metalloproteinase inhibitor, metastatic cancer, tumor microenvironment

## Abstract

The treatment of metastatic cancer is a great challenging issue throughout the world. Conventional chemotherapy can kill the cancer cells and, whereas, would exacerbate the metastasis and induce drug resistance. Here, a new combinatorial treatment strategy of metastatic cancer was probed via subsequentially dosing dual nanomedicines, marimastat‐loaded thermosensitive liposomes (MATT‐LTSLs) and paclitaxel nanocrystals (PTX‐Ns), via intravenous and intratumoral injection. First, the metastasis was blocked and cancer cells were locked in the tumor microenvironment (TME) by delivering the matrix metalloproteinase (MMP) inhibitor, MATT, to the tumor with LTSLs, downregulating the MMPs by threefold and reducing the degradation of the extracellular matrix. And then, the “locked” cancer cells were efficiently killed via intratumoral injection of the other cytotoxic nanomedicine, PTX‐Ns, along with no metastasis and 100% inhibition of tumor growth. This work highlights the importance of the TME's integrity in the chemotherapy duration. We believe this is a generalized strategy for cancer treatment and has potential guidance for the clinical administration.

## 1 INTRODUCTION

Irrespective of the vast advance in cancer treatment, metastasis emerging in the entire stage of cancer progression is still a major cause of cancer‐related death.^1^, ^2^ Particularly, breast cancer is a highly metastatic cancer and approximately 90% death of breast cancer patients was caused by the metastasis and resultant poor chemotherapy response.^3^ Undeniably, preventing metastasis plays an essential role in the treatment of metastatic breast cancer.^4^ Conventional chemotherapy can efficiently kill tumor cells, however, it facilitates the metastasis as well.^5^

Metastasis is closely linked with the tumor microenvironment (TME) which consists of non‐cancer cells, extracellular matrix (ECM), blood vessels, and lymphatics and is a sanctuary for cancer cells.^6^, ^7^ To kill the cancer cells efficiently, numerous formulations were designed to deliver the chemotherapeutic agents to the TME; nonetheless, the conventional formulation‐based chemotherapy resulted in destruction of the TME induced by the killing of non‐cancer cells like fibroblast and degradation of the ECM like collagen,^8^, ^9^, ^10^, ^11^ driving the cancer cells to escape from their broken “home (TME)” and exacerbating the metastasis. As a result, keeping the integrity of the TME would help inhibit the metastasis. The ECM—a major component of the TME, which is composed of a great number of macromolecules, such as proteins, glycoproteins, proteoglycans, and polysaccharides—is a critical scaffold of the TME and maintains its integrity.^12^ However, the ECM would be discomposed by matrix metalloproteinases (MMPs) in the TME.^7^ The MMPs are a type of zinc‐dependent proteases secreted by cancer cells predominantly and can degrade the ECM components, such as collagens, gelatins, fibronectins, and other related proteins, leading to TME's destroy.^7^, ^12^, ^13^ Accordingly, suppressing the MMPs is expected to keep the TME's integrity and then block the metastasis.

In our previous reports, we had demonstrated well that marimastat (MATT) was able to inhibit the MMPs and the resulted metastasis effectively via mimicking the substrate of the MMPs to work with MMPs in a competitive and reversible pattern.^14^, ^15^, ^16^ MATT is of low toxicity toward cancer cells. However, its combined use with cytotoxicity agents, for example, paclitaxel (PTX) which is a potent antitumor drug acting via targeting the tubulin, can compensate the drawback and treat metastatic cancer. Previously by assembling prodrug polymer‐PTX and MATT‐loaded nanoparticles, coloaded nanocomplexes were designed to treat metastatic cancer in a 4T1 tumor‐bearing model.^14^, ^15^ Although effective antitumor efficacy was demonstrated, the preparation of such complexes was too complicated to be characterized and to achieve the clinical transition.

Nanomedicines, including liposomes,^17^, ^18^, ^19^ nanocrystals,^16^, ^20^, ^21^, ^22^, ^23^ biomimetic nanoparticles,^24^, ^25^, ^26^, ^27^, ^28^ lipid drop‐based nanocapsules,^29^ polymer nanoparticles,^30^, ^31^, ^32^, ^33^, ^34^, ^35^ nanovaccines,^36^, ^37^ and so on, offer the promising potential to deliver the drug to the target site of interest with improved efficacy and reduced side effects.^38^, ^39^, ^40^ Dual nanomedicine‐based combinatorial therapy refers to coadministrating two separate nanomedicines. Compared with the single codelivery nanomedicine‐based combined treatment, this strategy features advantages in terms of delivering two drugs to different action sites, flexible administration by diverse dose/time schedule/routes, avoiding drug–drug interaction and, in particular, ease scale‐up and quality control, and being ready for regulation and commercialization as well, and so forth.^41^, ^42^, ^43^

Intravenous administration of chemotherapeutic agent is a commonly used approach to treat cancer in the clinic; meanwhile, serious toxicity to the healthy organs is not avoided due to high drug level in the circulation. By contrast, the intratumoral injection has significantly higher local drug concentration at the tumor site that can efficiently induce the apoptosis of cancer cells and compromise the drug resistance.^44^ Moreover, the local delivery decreases the systematic drug level dramatically and thus reduces the side effects^45^^.^

**Scheme 1:**
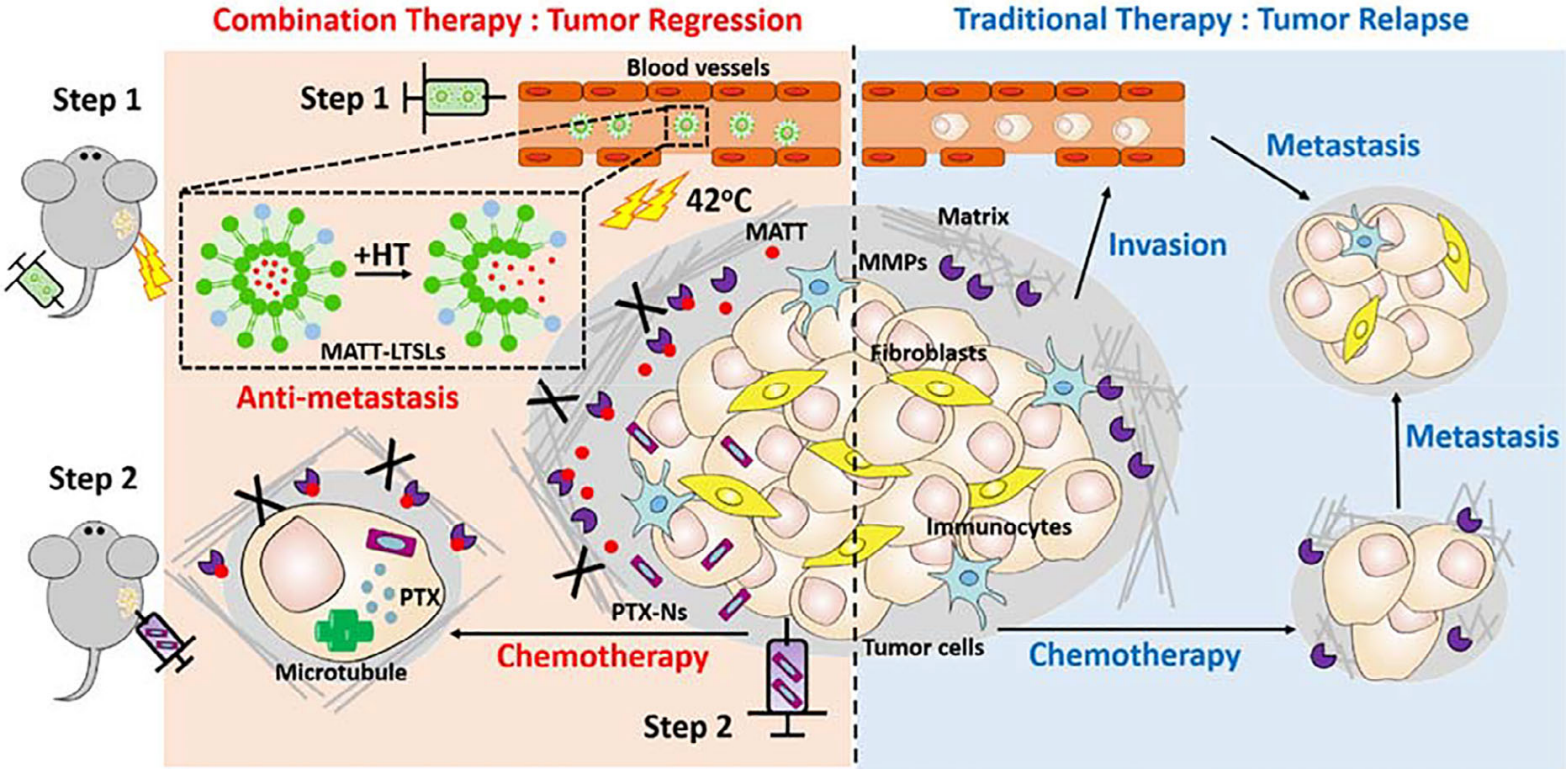
Design proposed an active mechanism for the sequential administration of MATT‐LTSLs and PTX‐Ns for targeting metastatic breast cancer. Step 1: MATT‐LTSLs are administrated via intravenous injection (i.v.). As MATT‐LTSLs penetrate into the tumor tissue, MATT is released from the LTSLs triggered by local heating and diffused into a tumor site. The released MATT inhibits the activity of MMPs via associating with MMPs in the tumor microenvironment (TME) and, therefore, maintains the integrity of the TME and secondarily suppresses the migration of cancer cells. Step 2: PTX‐Ns are administrated via intratumor injection, followed by entering the tumor cells, releasing the PTX in the cytoplasm and killing the tumor cells. Compared with the traditional chemotherapy, injecting MATT‐LTSLs in advance aims to inhibit the degradation of the extracellular matrix, block the tumor metastasis, and finally achieve tumor regression with high efficiency. MATT‐LTSLs, marimastat‐loaded thermosensitive liposomes; MMP, matrix metalloproteinase; PTX‐Ns, paclitaxel nanocrystals

Here, based on the dual nanomedicines, MATT‐loaded lysolipid‐containing thermosensitive liposomes (LTSLs) which the drug release was triggered by mild hyperthermia (HT) at 42°C and PTX nanocrystals (PTX‐Ns) featuring extremely high drug‐loading capacity, a new combinatorial treatment strategy of metastatic breast cancer was present. The two nanomedicines were administrated in a subsequential pattern. Unlike conventional combined‐therapy strategy, MATT‐LTSLs were intravenously injected, followed by HT treatment to stimulate MATT release in the TME to obtain an axial response—inhibit the expression of MMPs, suppress the ECM degradation, protect the TME integrity and “locked” the cancer cells in the TME. After that, the other cytotoxic nanomedicine, PTX‐Ns, were intratumorally injected to kill the “locked” tumor cells, thus achieving tumor elimination without metastasis (Scheme 1).

## 2 RESULTS

### 2.1 Nanoparticle preparation and characterization

**Figure 1:**
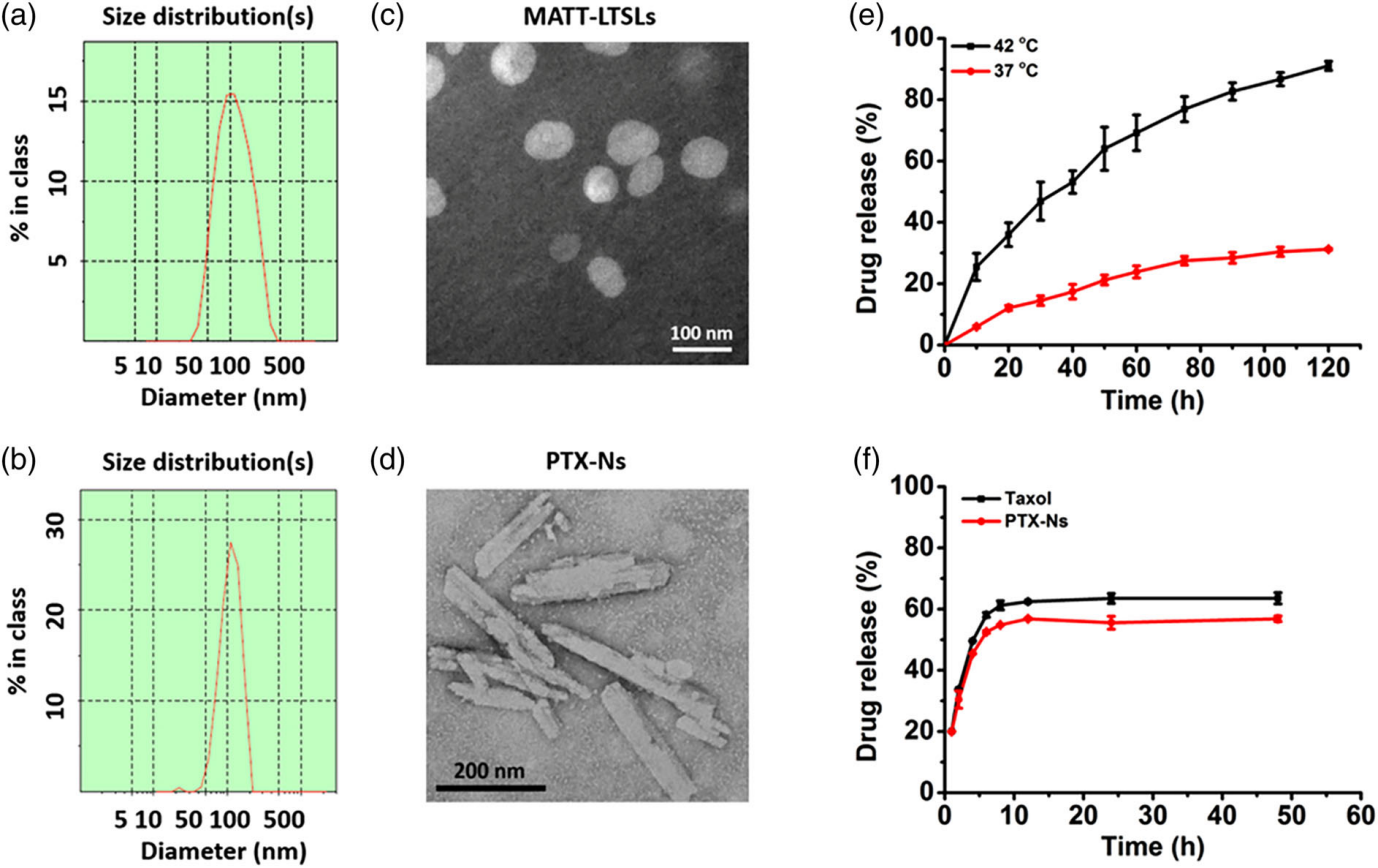
Characterization of nanoparticles. Size distribution and TEM images of (a, c) MATT‐LTSLs and (b, d) PTX‐Ns. (e) in vitro release profile of CF‐LTSLs at 42 or 37°C. (f) in vitro release profile of PTX‐Ns or Taxol at 37°C (mean ± *SD*, *n* = 3). MATT‐LTSLs, marimastat‐loaded thermosensitive liposomes; PTX‐Ns, paclitaxel nanocrystals; TEM, transmission electron microscope

LTSLs were prepared by a combined process of film hydration and ultrasonic treatment. The MATT‐loaded LTSLs (MATT‐LTSLs) had a particle size of 100 nm and a spherical morphology (Figure 1a,c), with encapsulation efficacy of approximately 60% assayed by high‐performance liquid chromatography method. PTX‐Ns were prepared using a precipitation‐ultrasonication method using β‐lactoglobulin (β‐LG) as a stabilizer to coat the PTX nanocrystals. PTX‐Ns exhibited as rod‐like nanoparticles with a length of 200 nm and a width of 30 nm (Figure 1b,d).

To detect the thermosensitivity of LTSLs, a fluorescence probe (CF) was encapsulated in LTSLs and temperature triggered drug release was measured at physical temperature and 42°C. At physical temperature of 37°C, the release of CF was less than 30% at 2 hr. In contrast at 42°C, CF was released up to 90% at 2 hr (Figure 1e). These results implied that LTSLs were stable in systemic circulation and could rapidly release their payload under HT treatment at 42°C in tumor site.

The release profile of PTX was carried out by dialysis method in pH 7.2 PBS simulated the pH of cell plasma. The release of PTX in PTX‐Ns was approximately 60% after 48 hr (Figure 1f), an indicator of sustained release of the drug in cells postinternalization.

### 2.2 Cellular uptake and cytotoxicity of PTX‐Ns

Flow cytometry displayed that the cellular uptake of fluorescein isothiocyanate (FITC)‐labeled PTX‐Ns was concentration‐ and time‐related (Figure S1 and Figure S2). Importantly, the intracellular fluorescence from the labeled nanoparticles was markedly greater than that of the free FITC, demonstrating an efficient uptake of the PTX‐Ns.

**Figure 2:**
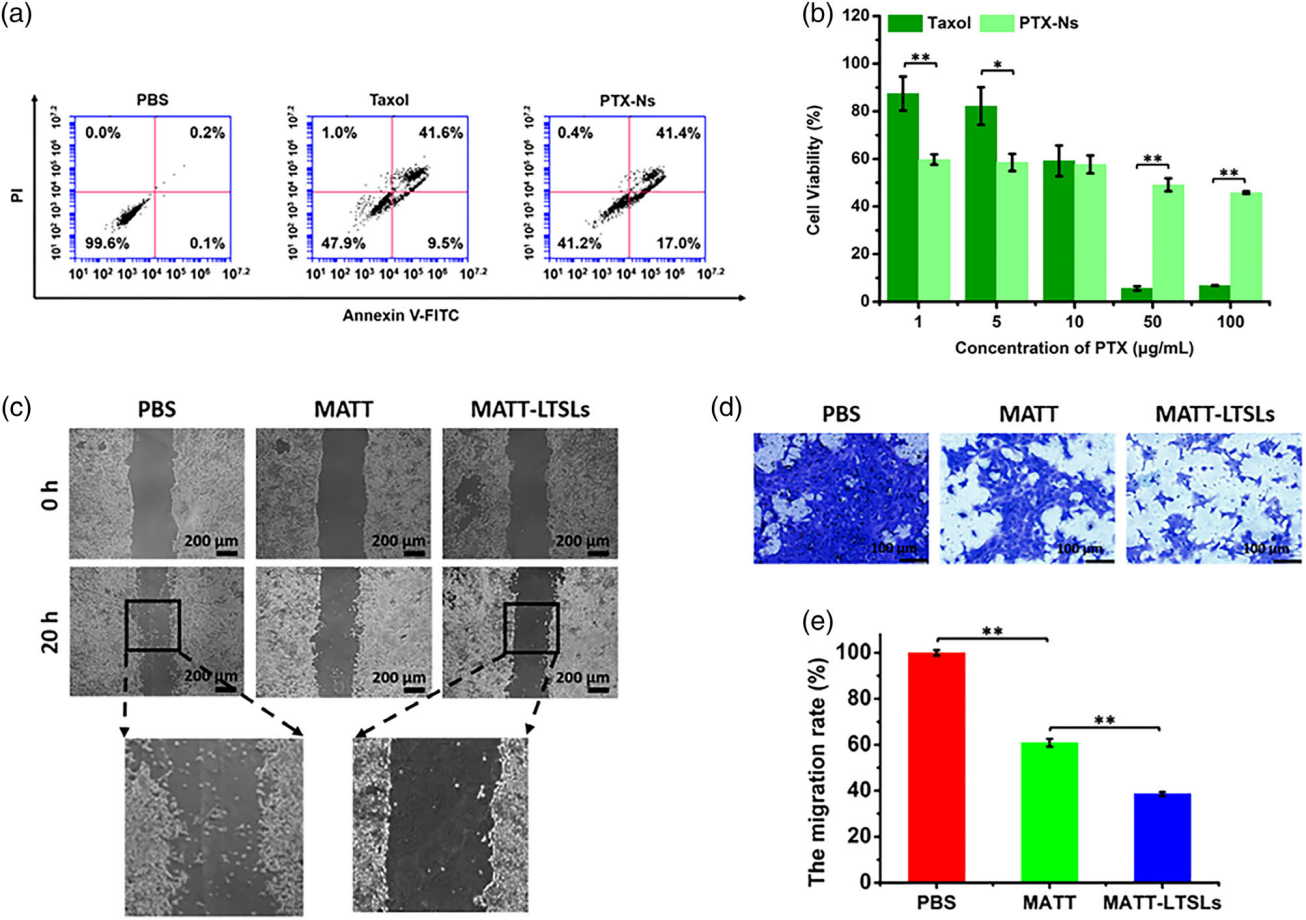
Antitumor efficacy in vitro. (a) The apoptosis rate of 4T1 cells was determined by Annexin V‐FITC/PI staining after 48‐hr incubation with PTX‐Ns or Taxol at a PTX concentration of 10 μg/ml at 37°C. The lower‐left, lower‐right, upper‐right, and upper left quadrants represented the viable, early apoptotic, late apoptotic, and dead cells, respectively. (b) Cell viability assessed by MTT after incubation with PTX‐Ns or Taxol for 48 hr at 37°C (mean ± *SD*, *n* = 5, ^*^ *p* < .05, ^**^ *p* < .01). (c) Cell migration evaluated by scratch wound‐healing assay after 20‐hr incubation with 10 μg/ml MATT at 37°C. (d) Cell invasion performed by a Matrigel® Transwell assay with a 24‐hr incubation with 10 μg/ml MATT at 37°C. Prior to incubation, MATT‐LTSLs were treated at 42°C for 1 hr to release the drug. The migrated cells were stained blue. (e) Quantification of the migration rate determined by optical density (OD) ratio at 570 nm (mean ± *SD*, *n* = 4, ^**^ *p* < .01). MATT‐LTSLs, marimastat‐loaded thermosensitive liposomes; MTT, 3‐(4,5)‐dimethylthiahiazo (‐z‐y1)‐3,5‐diphenyltetrazoliumromide; PTX‐Ns, paclitaxel nanocrystals

Apoptotic study in 4T1 cells indicated that PTX‐Ns allowed for apoptosis rate of 58%, greater than that from commercial formulation, Taxol, having a 51% rate (Figure 2a). 3‐(4,5)‐dimethylthiahiazo (‐zy1)‐3,5‐diphenyltetrazoliumromide (MTT) assay demonstrated that both of PTX‐Ns and Taxol showed dose‐related cytotoxicity (Figure 2b). These results demonstrated that PTX‐Ns killed cancer cells efficiently. Free MATT showed little cytotoxicity even at the dose of 50 μg/ml, whereas MATT‐LTSLs displayed significant cytotoxicity at high MATT concentrations (Figure S3A).

To examine the synergistic effects between MATT‐LTSLs and PTX‐Ns, we examined the cytotoxicity of the combined therapy (Figure S3B) and calculated the combination index (*CI*) by fitting the curve of a coefficient of a drug interaction. As depicted in Figure S3C, the *CI* values of the combined therapy at the PTX/MATT (wt/wt) ratio of 2:1 were less than 1 when the inhibition rate (*Fa*) ranged from 0.3 to 0.9, therefore demonstrated the potential synergism between the two nanomedicine.

### 2.3 In vitro inhibition of invasion and migration

To examine the antimetastatic ability of MATT‐LTSLs, wound healing assay, and transwell assay was conducted. The group treated with MATT‐LTSLs showed no cell movement across the scratch after a 20‐hr incubation compared with the PBS‐treated group (Figure 2c). PBS‐treatment did not inhibit the cell migration, displayed as large amounts of blue spots on the other side of the transwell membrane (Figure 2d,e). In contrast, the treatment with MATT formulations, particularly with MATT‐LTSLs, suppressed the migration significantly, with 60 and 40% reduction for MATT‐LTSLs and MATT, respectively. These results demonstrated MATT‐LTSLs inhibited cell invasion and migration significantly.

### 2.4 In vivo biodistribution of LTSLs

1,1′‐dioctadecyl‐3,3,3′,3′‐tetramethylindotricarbocyanine iodide (DiR), a near‐infrared probe, was loaded in LTSLs to assess the targeting ability of LTSLs. DiR‐LTSLs accumulated in tumor tissues with high efficacy in the duration of 24 hr (Figure S4A), compared with free probe DiR (Figure S4B). To further confirm the biodistribution of LTSLs, the main organs were harvested at 7, 12, and 24 hr postinjection. A strong fluorescence signal from DiR‐LTSLs in isolated tumors was observed as well (Figure S4C,D). Even at 24 hr after administration, the fluorescence signal in tumor remained strong, demonstrating LTSLs possessed the profound tumor‐targeting ability (Figure S4E).

Next, the penetration of LTSLs inside the tumor was further investigated. Colocalization of CF‐LTSLs with microvessels was indicated well (Figure S4F) and demonstrated that the LTSLs could penetrate inside the tumors and that the released payload in the nanoparticles had the potential to pass through the microvessels and locate in the TME.

### 2.5 Combined therapy in vivo

**Figure 3:**
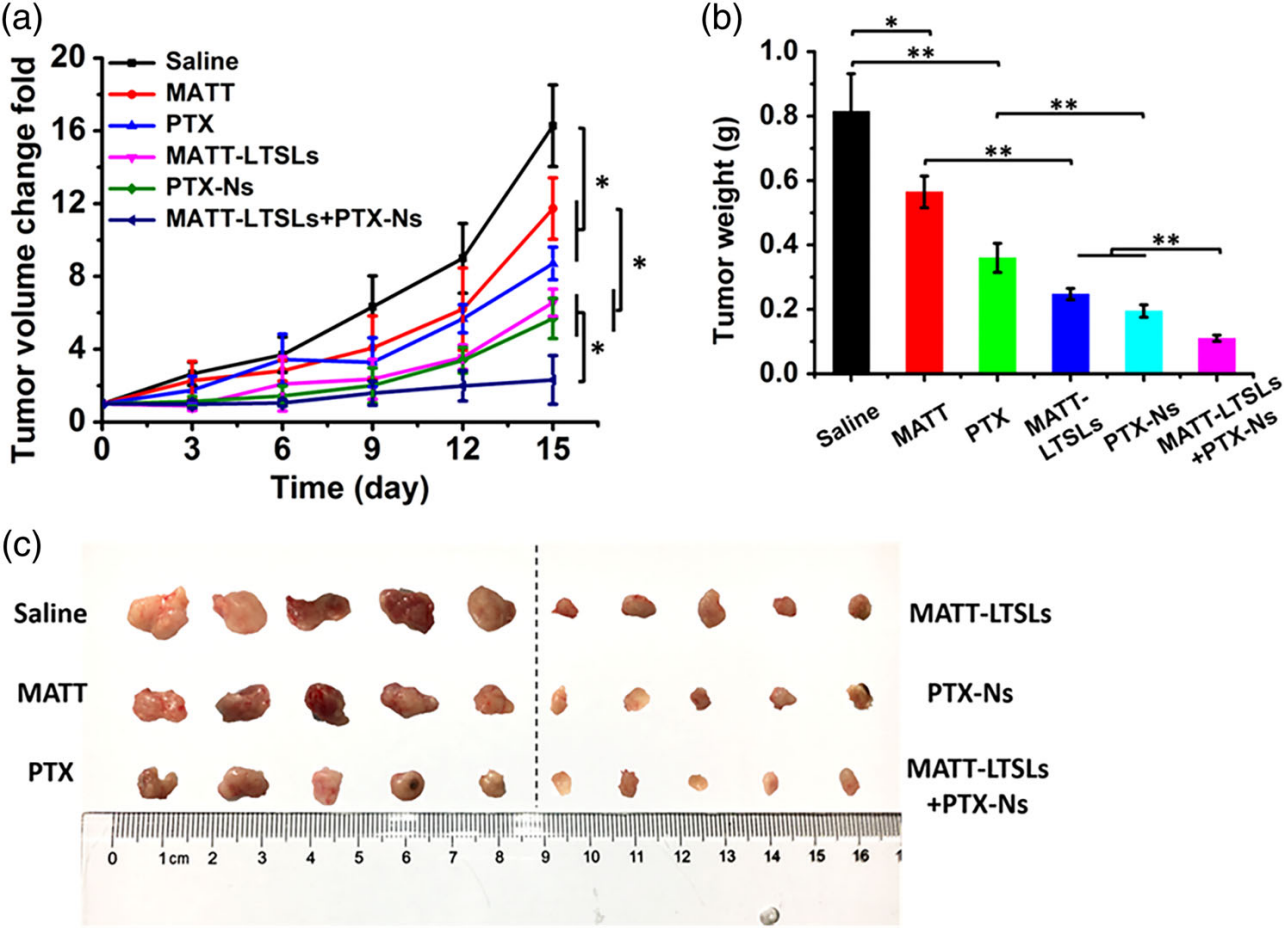
Antitumor efficacy in vivo in 4T1 tumor‐bearing Balb/C mice. Free PTX, MATT, and the same volume of saline were injected via the tail vein every 3 days at the dose of 10 mg/kg for PTX and 5 mg/kg for MATT, respectively. HT treatment was performed immediately by placing the tumor inside a water bath at 42°C for 45 min after the animals were injected with 10% chloral hydrate (wt/vol) at a dose of 400 mg/kg. For the group treated with dual nanomedicines, the mice were administered with MATT‐LTSLs in advance and, at 4 hr after HT treatment, were subjected to intratumor injection of PTX‐Ns. (a) Tumor volume change‐fold curves. (b) Tumor weight. The tumor was weighed on day 16 postinjection. (mean ± *SD*, *n* = 5, ^*^ *p* < .05, ^**^ *p* < .01). (c) Digital picture of tumors harvested on day 16 postinjection. HT, hyperthermia; LTSLs, lysolipid‐containing thermosensitive liposomes; MATT, marimastat; PTX‐Ns, paclitaxel nanocrystals

The in vivo antitumor efficacy in 4T1 tumor‐bearing mice treated with different formulation for 15 days was evaluated in terms of changes in tumor‐volume growth fold, tumor weight, tumor size, apoptosis, and proliferation of cancer cells in tumor (Figure 3 and Figure S5). The saline‐treated group exhibited a 17‐fold increase in tumor volume after five injections (Figure 3a). By contrast, treatment with MATT‐LTSLs or PTX‐Ns reduced the tumor volume by 1.6‐fold and 2‐fold compared to the free MATT or PTX treatment, respectively. Importantly, the treatment with the dual nanomedicines, MATT‐LTSLs plus PTX‐Ns, decreased the volume by twofold or eightfold compared with the treatment with single nanomedicine or saline. The tumor‐volume reduction was confirmed by measuring the weight and size of isolated tumors (Figure 3b,c). These results implied that the treatment with the dual nanomedicines inhibited the tumor growth profoundly.

Next, apoptosis and proliferation of cancer cells in the tumor were examined by TUNEL and Ki67 assay. The treatment with the dual nanomedicines exhibited an apoptotic rate of 60%, significantly greater than that of the treatment with single nanomedicine (Figure S5A,D), and displayed a proliferation rate of only 10%, 2–3 times less than that of the treatment with single nanomedicine (Figure S5B,E). Hematoxylin and eosin (H&E) staining assay further confirmed the examination of apoptosis and proliferation, displaying maximal necrosis in the group treated with the dual nanomedicines (Figure S5C).

### 2.6 Inhibition of angiogenesis and lung metastasis

**Figure 4:**
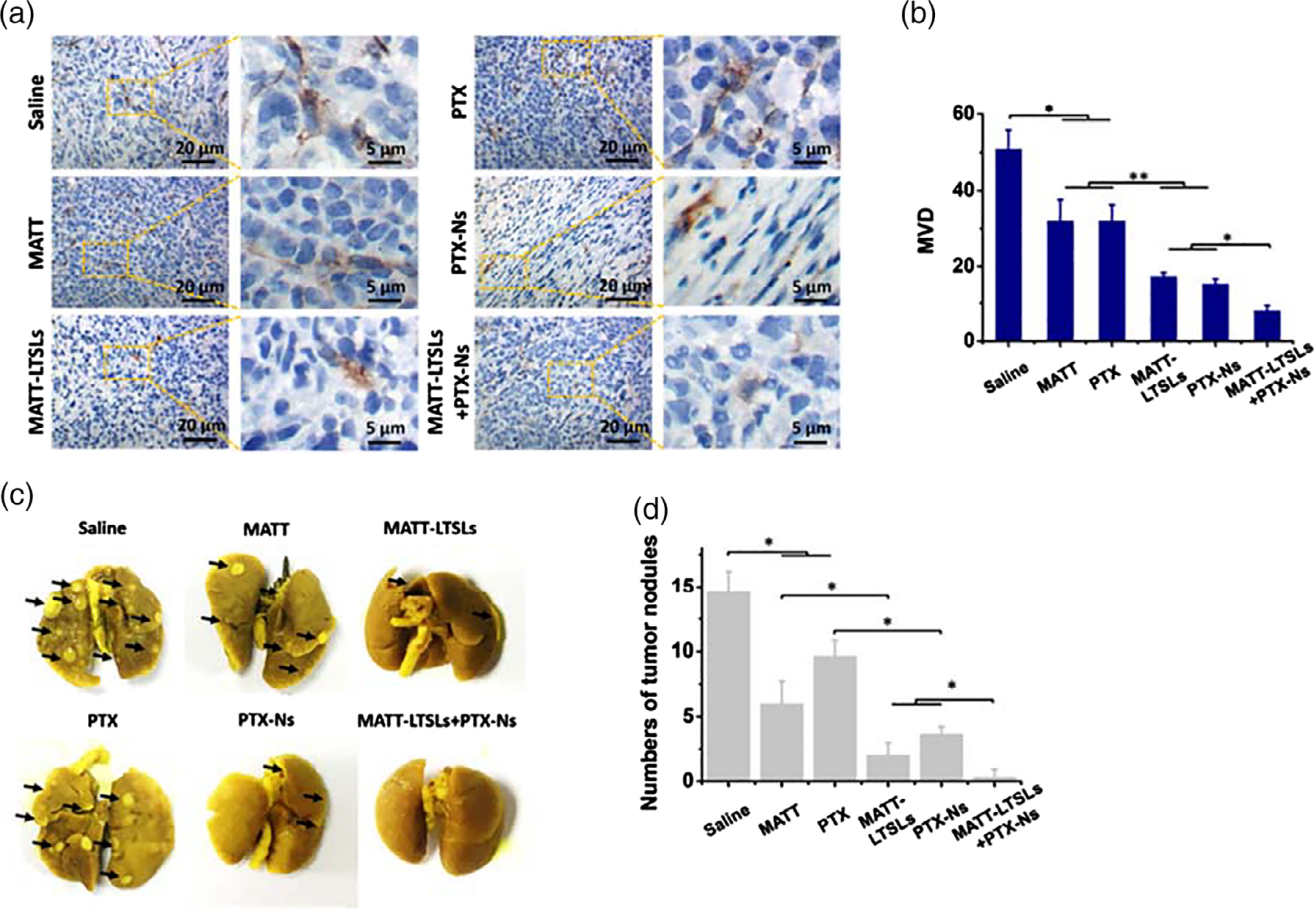
Inhibition of angiogenesis and lung metastasis in vivo. (a) Immunochemistry of CD31 staining for microvessels (brown) in tumor tissues collected from 4T1 tumor‐bearing Balb/C mice at Day 16 after treatment. (b) Quantitative analysis of microvascular density (MVD). MVD was quantified by five representative fields of cell nuclei under an optical microscope. (c) Digital images of lungs collected from 4T1 tumor‐bearing Balb/C mice at day 16 after treatment. The arrow indicated the tumor nodules on the lungs. (d) Quantitative analysis of tumor nodules on lungs by counting the numbers (mean ± *SD*, *n* = 3, ^*^ *p* < .05, ^**^ *p* < .01)

Angiogenesis is closely related with the metastasis.^14^, ^15^ Here, microvascular density (MVD) in the separated tumors collected at the end of treatment was investigated (Figure 4a,b). In the saline group, the MVD was 50 vessels per field. By contrast, the treatment with the single nanomedicine reduced the MVD by 2–3‐fold and, more importantly, the MVD further declined by approximately twofold after the dual nanomedicine treatment.

The 4T1 tumor is a highly metastatic breast cancer, predominantly metastasizing to the lung and secondarily to the liver.^4^, ^46^ Here, the study of lung metastasis was performed by counting the nodules on the lung isolated at the end of treatment (Figure 4c,d). The control group treated with saline exhibited 15 nodules on the lung. By contrast, the treatment with MATT and, in particular, with MATT‐LTSLs, reduced the number of tumor nodules to 6 and 2, respectively, being consistent well with the in vitro antimetastatic study described in Figure 2c–e. Most importantly, the dual nanomedicine‐treatment exhibited zero nodules.

Taken together, the dual nanomedicine treatment was able to markedly inhibit the angiogenesis and, especially, completely block the metastasis in the 4T1 tumor‐bearing metastatic model.

### 2.7 Inhibition of TME remodeling

#### 2.7.1 Inhibition of MMP expression and activity

**Figure 5:**
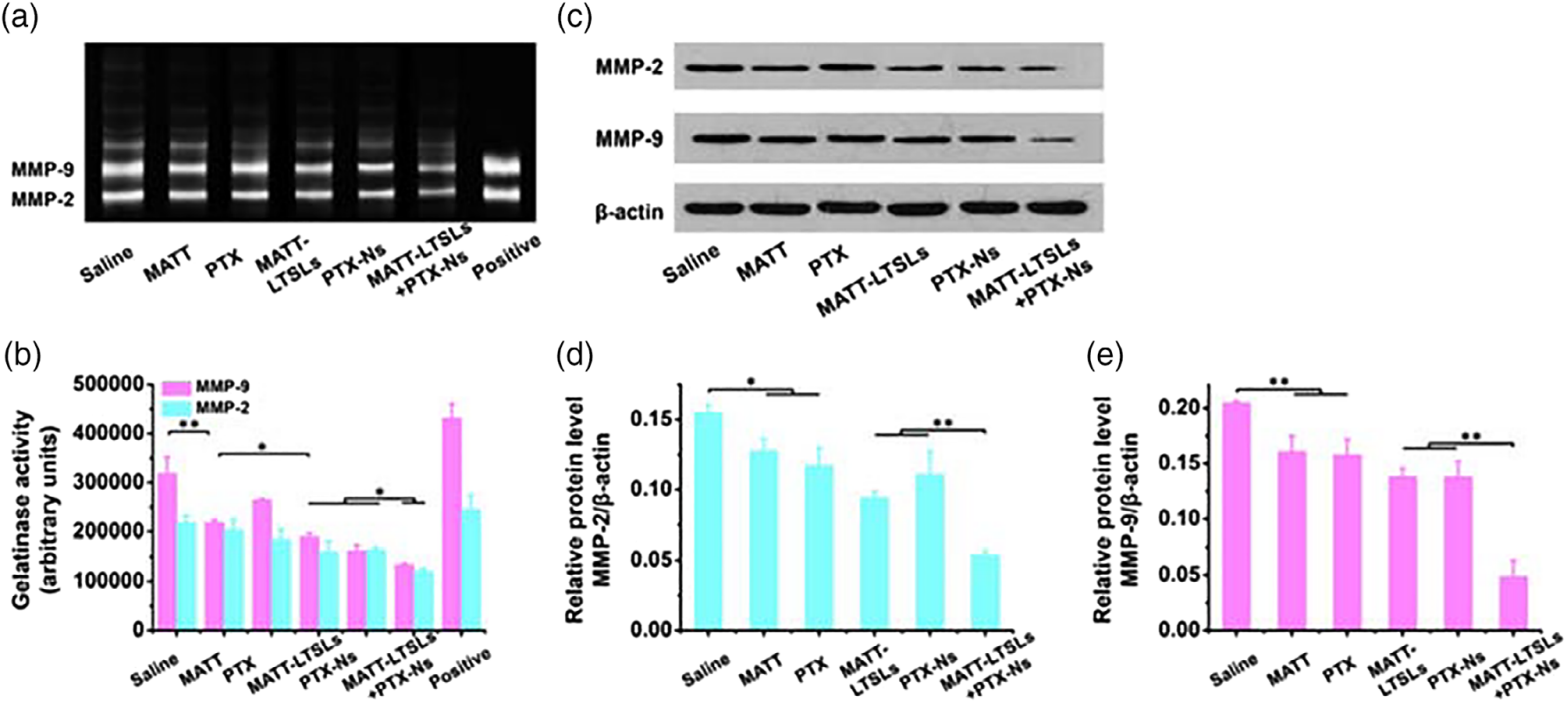
Inhibition of matrix metalloproteinase (MMPs) in vivo. (a) The activity of MMP‐2 and MMP‐9 was measured by gelatin zymography. Clear bands indicated gelatin degradation by MMPs and the activity of MMPs. (b) Quantitative analysis of MMP activity from zymograms using a computer analysis program. (c) Western blot analysis of MMP‐2 and MMP‐9 expression of tumors. Quantitative analysis of (d) MMP‐2 and (e) MMP‐9 expression. β‐actin was used as a loading control. Tumors used in these experiments were collected from 4T1 tumor‐bearing Balb/C mice at Day 16 after treatment (mean ± *SD*, *n* = 3, ^*^ *p* < .05, ^**^ *p* < .01)

MMP‐2 and MMP‐9, also known as gelatinases, were highly expressed in breast cancer and play an essential role in metastasis and invasion.^7^, ^12^, ^13^ In this study, the expression and activity of the two MMPs were evaluated by western blot assay and gelatin zymography, respectively, to probe the mechanism of antimetastasis. The activity of MMPs was reduced by all formulations compared with the control saline (Figure 5a,b); however, the maximal reduction was observed in the dual nanomedicine‐treated group, with 1.5‐and 1‐fold decrease for MMP‐9 and MMP‐2, respectively. Western blot assay and quantified analysis indicated the expression of MMPs was decreased by 2.5‐fold and 4‐fold for MMP‐2 and MMP‐9, respectively, after treatment with the dual nanomedicines compared with the control saline (Figure 5c–e). Indeed, besides the dual nanomedicines, the MATT‐LTSLs displayed significant downregulation of the MMPs as well, demonstrating the potent delivery of MATT using LTSLs. In short, the dual nanomedicine‐based approach allowed for profound inhibition of MMPs' activity and expression. Also, compared with using MATT‐LTSLs or PTX‐Ns alone, the dual nanomedicine‐treatment could suppress the MMPs with higher efficacy and, as a result, demonstrated a potential synergistic effect between the two nanomedicines.

#### 2.7.2 Inhibition of ECM degradation

**Figure 6:**
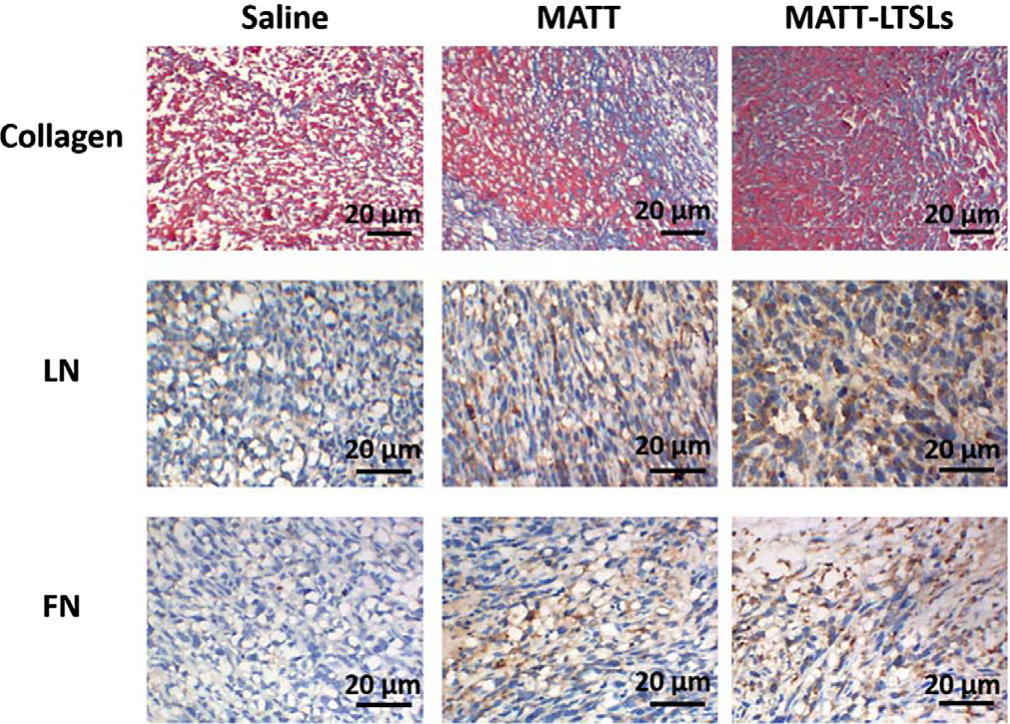
Inhibition of extracellular matrix (ECM) degradation in vivo. The expression of collagen was analyzed by Masson trichrome staining. The collagen was stained blue. Laminin (LN), fibronectin (FN) were analyzed by immunohistochemical staining. The LN and FN proteins were stained brown. Tumor tissues for these experiments were collected from 4T1 tumor‐bearing mice on Day 16 post‐treatment

The ECM is a supportive scaffold of the TME and is critical to maintaining the integrity of TME. However, most of the ECM, such as collagen, laminin (LN), and fibronectin (FN), is MMPs's substrate^7^, ^12^; and accordingly, the highly expressed MMPs in tumor would discompose the ECM and thus jeopardize the TME's integrity. The experimental results present in Figure 5 had demonstrated profound MMP inhibition via MATT‐LTSL‐treatment. Therefore, we hypothesized that the MATT formulation‐treatment could reduce the ECM degradation by MMPs in the tumor. As shown in Figure 6, the treatment with MATT and, especially, with MATT‐LTSLs upregulated the collagen expression measured by Masson Trichrome staining in comparison with the treatment with the saline control. The expression of the other two ECMs, LN, and FN, detected by immunochemistry staining was increased as well after treatment with MATT formulations. Compared with free MATT, the MATT‐LTSLs allowed for higher expression of the three ECMs. Overall, these results demonstrated that the administration of MATT‐LTSLs was able to suppress the degradation of ECM and thereby benefit the maintenance of the TME's integrity.

## 3 DISCUSSION

MATT is able to inhibit the metastasis, but its efficient delivery to the tumor site is critical. MATT was used in clinical study for tumor therapy 20 years ago; however, it failed in the Phase III study due to the modest efficacy and the cumulative toxicity of inflammation and musculoskeletal pain.^47^, ^48^ After that, the exploitation of the drug was terminated. Surprisingly, recent studies unveiled that the drug could inhibit the metastasis,^14^, ^15^ alleviate the inflammation,^49^ and regulate the immune system^50^ via inhibition of MMPs. Indeed, our previous reports indicated the drug blocked the metastasis significantly.^14^, ^15^, ^16^ On the other side, we found that the inhibition efficacy in metastasis from nanoparticle formulation was markedly different from that of the free drug, despite that their discrepancy of MMP inhibition in vivo was less than 20% (Figure 5).^16^ In this study, MATT‐LTSLs enabled threefold of metastasic nodules less than the free MATT (Figure 4c,d), being in line with our previous reports.^14^, ^15^, ^16^ Therefore, we speculate that abundant MMPs exist in the tumor site; and as a result, effective delivery of MATT to the tumor site with carriers is indispensable. The use of nanoparticles is possible to overcome the drawbacks of MATT but additional work is needed.

The “locked” cancer cells were more sensitive to chemotherapy. Undeniably, chemotherapy is the first choice for patient with cancer for its extremely low cost. Nevertheless, the chemotherapy is a double‐edged sword that can kill the cancer cells and, meanwhile, would induce metastasis and drug resistance, rendering most of the patient ending with chemotherapy‐response failure and death^51^; and also, the metastasis which occurs in the entire stage of cancer development would be further exacerbated by the broken TME by chemotherapy.^11^, ^52^, ^53^ Here, we uncovered poor response of 4T1 cells to the single treatment with free PTX or PTX‐Ns. In contrast, the administration of MATT formulation followed by PTX‐Ns displayed significant enhancement in apoptosis and inhibition of proliferation and tumor growth (Figure 3 and Figure S4). Because of the staying of TME's integrity by MATT, the metastasis was markedly blocked, an indicator that the cancer cells were fixed well in the TME (Figures 4 and 6). These results demonstrated that steady‐state cancer cells are more sensitive to chemotherapy. This finding can provide a guide for cancer therapy in the clinic.

The 4T1 cancer model is highly metastatic cancer. Subsequentially dosing the dual nanomedicines, MATT‐LTSLs and PTX‐Ns, via different routes could treat metastatic cancer with high efficacy. The two nanomedicines feature easy preparation, high payloads, and well‐known tumor‐targeting ability, therefore allowing the strategy for ready clinical translation. We believe this is a universal strategy that can be adapted to treat other metastatic diseases as well.

Although PTX‐Ns‐induced apoptosis with higher efficacy compared with free PTX, however, this nanomedicine exhibited lower cytotoxicity at high concentrations. Our previous reports also displayed similar results.^54^, ^55^, ^56^ Furthermore, combined use with other drugs, such as gene and protein, made this enhancement more profound.^54^, ^55^ The previous report indicated that internalized PTX‐Ns started to dissolve at 10 hr postinternalization, and therefore demonstrating sustained PTX release over time in cells.^57^ Consequently, the sustained release might help the chemotherapeutic agent loaded in nanomedicines kill the cancer cells. Indeed, additional experiments are needed to study the potential mechanism.

## 4 CONCLUSION

In summary, we have demonstrated a generalized strategy based on dual nanomedicines administered via a subsequential pattern for the targeted combination treatment of highly metastatic cancer such as breast cancer. Via first targeting delivery of the MMP inhibitor, MATT, to the tumor with LTSLs and the resultant maintenance of the TME's integrity, the metastasis is blocked and cancer cells are locked in the TME; and subsequently, the other cytotoxic nanomedicine, PTX‐Ns, is intratumorally injected and kill the “locked” cancer cells efficiently, exhibiting as zero metastasis to the lung and almost 100% inhibition of tumor growth. This work highlights the potential importance of keeping the TME's integrity in the chemotherapy duration. Additionally, owing to the facile preparation of the nanomedicines with high drug‐loading capacity, well‐known biocompatibility and the readily dosing, the strategy present here has promising potential for clinical transition and can be applied to treating other metastatic diseases as well.

## 5 MATERIALS AND METHODS

### 5.1 Materials

PTX was purchased from Yew Biotechnology Co., Ltd. (Jiangsu, China). Taxol (marked product of PTX) was purchased from Bristol‐Myers Squibb Investment Co., Ltd. (Shanghai, China). MATT was purchased from Nanjing Adooq Co., Ltd. (Jiangsu, China). β‐LG (90% purity), CF, FITC, and MTT were obtained from Sigma‐Aldrich Co., Ltd. (St. Louis, MO). DiR was purchased from Biotium, Inc. (Hayward, CA). 1,2‐dipalmitoyl‐DL‐alpha‐phosphatidylcholine (DPPC), 1,2‐distearoyl‐sn‐glycero‐3‐phosphoethanolamine‐*N*‐[methoxy(polyethylene glycol)‐2000] (DSPE‐PEG2000) were purchased from Lipoid (Germany). 1‐stearoyl‐2 hydroxy‐sn‐glycero‐3‐phosphocholine (1‐StePc) was purchased from Shanghai AVT Pharmaceutical Technology Co., Ltd. (Shanghai, China). 4T1 cell line was purchased from Nanjing KeyGEN Biotech Co., Ltd. (Nanjing, China). Fetal bovine serum (FBS), RPMI‐1640, penicillin–streptomycin solution and trypsin were obtained from Wisent, Inc. (Nanjing, China). Annexin V‐FITC/PI staining kit was obtained from the Beyotime Institute of Biotechnology (Haimen, China).

BALB/c mice (18–20 g) were provided by the College of Veterinary Medicine Yangzhou University (Yangzhou, China). The animals acquired care that followed the Principles of Laboratory Animal Care and the Guide for the Care and Use of Laboratory Animals. Experiments followed the protocol approved by the China Pharmaceutical University Institutional Animal Care and Use Committee.

### 5.2 Cell cultures

4T1 cells were cultured in RPMI 1640 medium containing 10% FBS and 1% penicillin streptomycin solution, and incubated at 37°C under 5% CO_2_. The cells were harvested with trypsin and cell suspensions were prepared for further experiments.

### 5.3 Nanoparticle preparation and characterization

MATT‐LTSLs were prepared as described in our previous report.^15^ Briefly, DPPC: 1‐StePc: DSPE‐PEG2000 at a mass ratio of 86:10:4 were dissolved in a solution of chloroform: methanol = 3:1 (vol/vol), followed by drying at 45°C under vacuum, hydration with pH 6.5 PBS at 45°C for 40 min, treatment with miniature ultrasonic probe (20–25 kHz, Scientz Biotechnology Co., Ltd., Ningbo, China), passing through a 0.22‐μm membrane filter, and ultrafiltration. The dye‐loaded LTSLs were prepared by a similar process.

For the preparation of PTX‐Ns, 1 ml acetone containing 10 mg PTX was mixed with 5 ml β‐LG solution (1 mg/ml in water) under stirring conditions, treated with an ultrasonic probe for 15 min at 400 W and dried at vacuum.^54^, ^58^ The dye‐labeled PTX‐Ns were prepared by a similar method, except that the dye DMSO was mixed with the PTX acetone in advance of adding the stabilizer solution.

The diameter of nanoparticles was measured according to the dynamic light scattering principle by a 90Plus Particle Size Analyzer (Brookhaven Instruments, Holtsville, NY).

Transmission electron microscope (TEM) was carried out to examine the morphology of nanoparticles. The diluted samples were dropped on a copper mesh and kept for 5 min. After removing the extra sample, the copper mesh was stained by 2% (wt/wt) phosphotungstic acid for 1 min, dried at room temperature and imaged by a JEM‐1230 TEM (Tokyo, Japan).

### 5.4 Thermosensitivity and drug release

To determine the thermosensitivity of LTSLs, CF loaded LTSLs (CF‐LTSLs) were prepared. The release of CF was carried out by a dialysis method in a shaker (SHA‐C, Jintan, China) at a speed of 100 rpm. The samples (1 ml) were added into a dialysis bag (MWCO 8,000–14,000 Da) and incubated in 30 ml PBS solution (pH 6.5) at 37 or 42°C, and sampled with the replacement of fresh medium at the predetermined time points. The released CF was determined by a microplate reader (POLARstar Omega, Germany) at the excitation wavelength and an emission wavelength of 492 and 515 nm, respectively.

The drug release from Taxol and PTX‐Ns was tested by a dialysis method under a shaker at 37°C as well. The detailed process and PTX assay were described in our previous report.^54^

### 5.5 Flow cytometry

4T1 cells (1 × 10^5^) seeded in 12‐well plates for 48 hr were incubated with dye‐labeled nanoparticles at 37°C. After that, the cells were harvested, washed with cold PBS and resuspended in 500 μl of PBS for flow cytometry analysis (Accuri C6, BD).

### 5.6 Confocal imaging

Cells (2 × 10^5^) seeded on 12 mm round glass coverslip in advance for cell attachment were incubated with dye‐labeled nanoparticles in serum‐free medium for 2 hr at 37°C, washed with cold PBS three times, reacted with 4% paraformaldehyde and stained with DAPI for 10 min, and finally observed with a LSM700 confocal laser scanning microscopy (CLSM, Carl Zeiss, Germany).

### 5.7 In vitro cytotoxicity

4T1 cells were seeded in 96‐well plates at a density of 5 × 10^3^ cells per well for 24 hr. Then the cells were incubated with PTX‐Ns, Taxol, MATT‐LTSLs, MATT, MATT+PTX, MATT‐LTSLs+PTX‐Ns at 37°C. For the single drug therapy, the cells were treated for 48 hr with the drug formulations. And for combination therapy, the cells were first treated with MATT formulations for 4 hr and, then, incubated with PTX formulations for another 48 hr. The ratio of MATT and PTX was 1:2 (wt/wt). MATT‐LTSLs were heated at 42°C in a water bath for 45 min before treatment with cells. After incubation, cells were cultured with MTT (1 mg/ml) for 4 hr, followed by the removing of culture medium, the addition of 150 μl DMSO and absorbance measurement at the wavelength of 570 nm using a microplate reader (Multiskan FC, Thermo Fisher Scientific). Additionally, based on the cytotoxicity, the coefficient drug interaction between the two nanomedicines was analyzed by the CompuSyn software.

The cell apoptosis was treated by Annexin V‐FITC/PI apoptosis detection kits followed standard protocol and detected by flow cytometry (Accuri C6, BD).

### 5.8 Wound healing test and transwell

4T1 cells (1 × 10^5^) were seeded in 12‐well plates for a cover density of 70–80% reached, followed by scratch making using a 200‐μl pipette. After that, cells were cultured in a serum‐free medium and treated with MATT formulations at a MATT concentration of 5 μg/ml. MATT‐LTSLs were preheated at 42°C in a water bath for 1 hr. Images were taken at 0 and 20 hr postscratching with an optical microscope (Olympus IX53, Japan).

Transwell assays were performed in 24‐well Transwell chambers with an 8‐μm pore (Corning Life Sciences, Inc.), as described in our reports.^15^, ^55^ In brief, 4T1 cells seeded at a density of 1 × 10^5^ cells per well in the upper chamber for 24 hr, were incubated with 200 μl of serum‐free medium and treated with MATT formulations at a MATT concentration of 1 μg/ml for 4 hr at 37°C. MATT‐LTSLs were preheated at 42°C in a water‐bath for 1 hr before incubation with cells. Subsequently, the cells in the upper chamber were removed with a cotton swab and, meanwhile, the cells attached on the lower surface of the filter were fixed with 4% paraformaldehyde, stained with 0.1% crystal violet for 10 min and washed. The stained cells were imaged by optical microscopy (Olympus IX53, Japan). Crystal violet was dissolved in 33% acetic acid and the optical density ratio was measured at the wavelength of 570 nm using a microplate reader (Multiskan FC, Thermo Fisher Scientific).

### 5.9 In vivo imaging and biodistribution

First, 4T1 cells were suspended in saline with the density of 1 × 10^7^ cells/ml and injected to the upper back of BALB/c mice (female, 20–22 g) subcutaneously with a volume of 0.1 ml. After the tumor reached the volume of 400–500 mm^3^, DiR‐labeled nanoparticles (200 μl) were injected into the mice via tail vein at a DiR dose of 0.5 mg/kg. At specific time points after injection, the mice were anesthetized by isoflurane for imaging in an in vivo imaging system (IVIS Spectrum, PerkinElmer). At 7, 12, and 24 hr after injection, the mice were sacrificed to sample the major organs for ex vivo imaging.

The intratumoral distribution of LTSLs was studied via the injection of CF‐labeled LTSLs at a CF dose of 1 mg/kg. Briefly, at 2 hr postinjection, the tumors were isolated, sectioned, stained with Cy7‐labeled anti‐mouse CD31 antibody (Abcam, Britain), and observed with CLSM.

### 5.10 In vivo antitumor activity

Six groups (*n* = 5) of 4T1 tumor‐bearing mice were injected with 0.2 ml of saline, free MATT, MATT‐LTSLs, and Taxol via tail vein, and with 0.1 ml of PTX‐Ns via intratumoral injection, respectively. The group treated with dual nanomedicines were first injected 0.2 ml of MATT‐LTSLs and, 6 hr later, were injected intratumorally with 0.1 ml PTX‐Ns. HT treatment for MATT‐LTSLs was performed by placing the tumor tissue in a water bath at 42°C for 45 min after the injection. The body weight and tumor volume were recorded and tumor volume change fold was calculated every 3 days during the treatment period. At the end of treatment, the mice were sacrificed to collect tumors and lung tissues for further study.

Cell apoptosis and proliferation in the tumor were detected by TUNEL and Ki67 kits (Beyotime, China), respectively. H&E staining was performed to observe the pathological changes of tumor tissues. Angiogenesis in the tumor was evaluated by an index of MVD. Metastasis in vivo was evaluated by counting the tumor nodules on the lungs. The expression and activity of MMPs in tumors were assessed by western blot and gelatin zymography, respectively. Detailed experimental methods were present in our previous report.^15^

Immunochemistry staining and Masson Trichrome staining for the sectioned tumor were carried out to examine the integrity of TME. Briefly, tumor tissues were fixed in 4% paraformaldehyde, embedded in paraffin to prepare sections for immunohistochemistry test according to the standard protocol. A fibronectin (FN) antibody (Abcam, Britain) and a laminin (LN) antibody (BOSTER, China) were used as primary antibody respectively to detect the component of FN and LN. For Masson Trichrome staining, the sectioned‐tumor was deparaffinized, rehydrated, stained with a Masson Trichrome kit following the manufacturer's direction and observed under an optical microscope (B1‐330, Motic, China).

### 5.11 Statistical analysis

Data were presented as means ± *SD* one‐way analysis of variance was performed to assess the statistical significance of the differences between samples. *p* < .05 was considered significant.

## Supporting information


**Figure S1. Cellular uptake of FITC/PTX‐Ns in a concentration‐dependent pattern.** Fluorescence intensity quantification and flow‐cytometry determination after incubation with (A, C) FITC/PTX‐Ns and free FITC (B and D, control) at different concentrations at 37°C for 4 h. (mean ± S.D., *n* = 3, **p < 0.01).
**Figure S2. Cellular uptake of FITC/PTX‐Ns in a time‐dependent pattern.** (A) Fluorescence intensity quantification and (B, C) flow‐cytometry determination after incubation with FITC/PTX‐Ns or free FITC (C, control) at a FITC concentration of 500 ng/mL at 37°C for various durations. (mean ± S.D., *n* = 3, **p < 0.01).
**Figure S3. Combination Index (CI) Calculation**. Cell cytotoxicity of 4 T1 treated with (A) MATT formulations and (B) combined formulations of free drugs and nanomedicine for 48 h at 37°C (mean ± S.D., *n* = 5, **p < 0.01). (C) The curve of coefficient of drug interaction between MATT‐LTSLs and PTX‐Ns. *CI* was calculated by CompuSyn software. *CI* > 1, *CI* < 1, *CI* = 1 indicate antagonistic effect, synergistic effect and addictive effect, respectively. The two nanomedicines showed the synergistic effect when the inhibition rate is between 0.3 and 0.9.
**Figure S4. Biodistribution of DiR‐labeled LTSLs in 4 T1 tumor‐bearing Balb/C mice.** (A, B) Image of whole body at different time points after administration of (A) DiR‐LTSLs and (B) free DiR through tail vein at the DiR dose of 0.5 mg/kg (*n* = 3). (C, D) ex vivo image of major tissues collected at 7 h, 12 h, 24 h post injection of (C) DiR‐LTSLs and (D) free DiR. (E) Fluorescence quantification of different tissues (mean ± S.D., *n* = 3). (F) The colocalization of CF‐LTSLs (green) with microvessels stained with Cy7‐labeled CD31 antibody (Red). The nucleus were stained with DAPI (blue). The yellow fluorescence spots indicated the colocalization. Scale bar of the enlarged view, 5 μm.
**Figure S5. Histological study.** (A) TUNEL, (B) Ki67 and (C) H&E staining of sectioned tumor collected from 4 T1 tumor‐bearing Balb/C mice on day 16 after treatment. Quantitative analysis of tumor cell (D) apoptosis rate and (E) proliferation rate. Cell apoptosis and proliferation rate were quantified by five representative fields of cell nuclei under an optical microscope (mean ± S.D., *n* = 5, **p < 0.01). In H&E analysis, nuclei are stained blue, while extracellular matrix and cytoplasm are stained red.
