# Supplementary material for: “Locked” cancer cells are more sensitive to chemotherapy
Source: Bioeng Transl Med. 2019 Jun 10;4(2):e10130. doi: 10.1002/btm2.10130 (PMC6584094; doi:10.1002/btm2.10130)
Supplement: Supplementary file 1 — Figure S1. Cellular uptake of FITC/PTX‐Ns in a concentration‐dependent pattern. Fluorescence intensity quantification and flow‐cytometry determination after incubation with (A, C) FITC/PTX‐Ns and free FITC (B and D, control) at different concentrations at 37°C for 4 h. (mean ± S.D., n = 3, **p < 0.01). Figure S2. Cellular uptake of FITC/PTX‐Ns in a time‐dependent pattern. (A) Fluorescence intensity quantification and (B, C) flow‐cytometry determination after incubation with FITC/PTX‐Ns or free FITC (C, control) at a FITC concentration of 500 ng/mL at 37°C for various durations. (mean ± S.D., n = 3, **p < 0.01). Figure S3. Combination Index (CI) Calculation. Cell cytotoxicity of 4 T1 treated with (A) MATT formulations and (B) combined formulations of free drugs and nanomedicine for 48 h at 37°C (mean ± S.D., n = 5, **p < 0.01). (C) The curve of coefficient of drug interaction between MATT‐LTSLs and PTX‐Ns. CI was calculated by CompuSyn software. CI > 1, CI < 1, CI = 1 indicate antagonistic effect, synergistic effect and addictive effect, respectively. The two nanomedicines showed the synergistic effect when the inhibition rate is between 0.3 and 0.9. Figure S4. Biodistribution of DiR‐labeled LTSLs in 4 T1 tumor‐bearing Balb/C mice. (A, B) Image of whole body at different time points after administration of (A) DiR‐LTSLs and (B) free DiR through tail vein at the DiR dose of 0.5 mg/kg (n = 3). (C, D) ex vivo image of major tissues collected at 7 h, 12 h, 24 h post injection of (C) DiR‐LTSLs and (D) free DiR. (E) Fluorescence quantification of different tissues (mean ± S.D., n = 3). (F) The colocalization of CF‐LTSLs (green) with microvessels stained with Cy7‐labeled CD31 antibody (Red). The nucleus were stained with DAPI (blue). The yellow fluorescence spots indicated the colocalization. Scale bar of the enlarged view, 5 μm. Figure S5. Histological study. (A) TUNEL, (B) Ki67 and (C) H&E staining of sectioned tumor collected from 4 T1 tumor‐bearing Balb/C mice on day 16 [file BTM2-4-na-s001.docx]

**Supplementary materials**

**"Locked" Cancer Cells Are More Sensitive to Chemotherapy**

*Yaqi Lyu, Qingqing Xiao, Yi Li, Yubing Wu, Wei He *, Lifang Yin **

Department of Pharmaceutics, School of Pharmacy, China Pharmaceutical University, Nanjing, 210009, PR China

*Corresponding authors to Yin, LF or He, W

Department of Pharmaceutics, School of Pharmacy, China Pharmaceutical University

E-mail: 1019940752@cpu.edu.cn, weihe@cpu.edu.cn


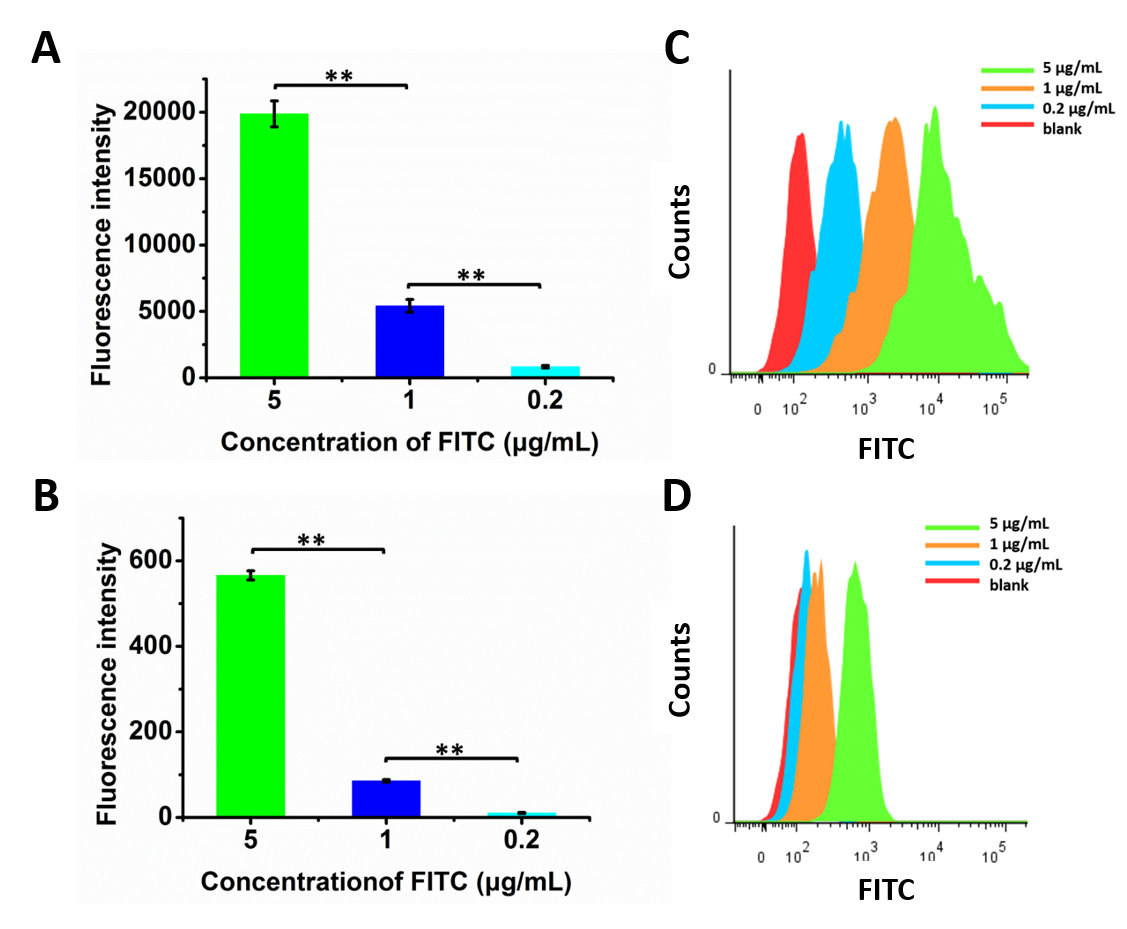


**Figure S1. Cellular uptake of FITC/PTX-Ns in a concentration-dependent pattern.** Fluorescence intensity quantification and flow-cytometry determination after incubation with (A, C) FITC/PTX-Ns and free FITC (B and D, control) at different concentrations at 37 °C for 4 h. (mean ± S.D., *n* = 3, ***p* < 0.01).


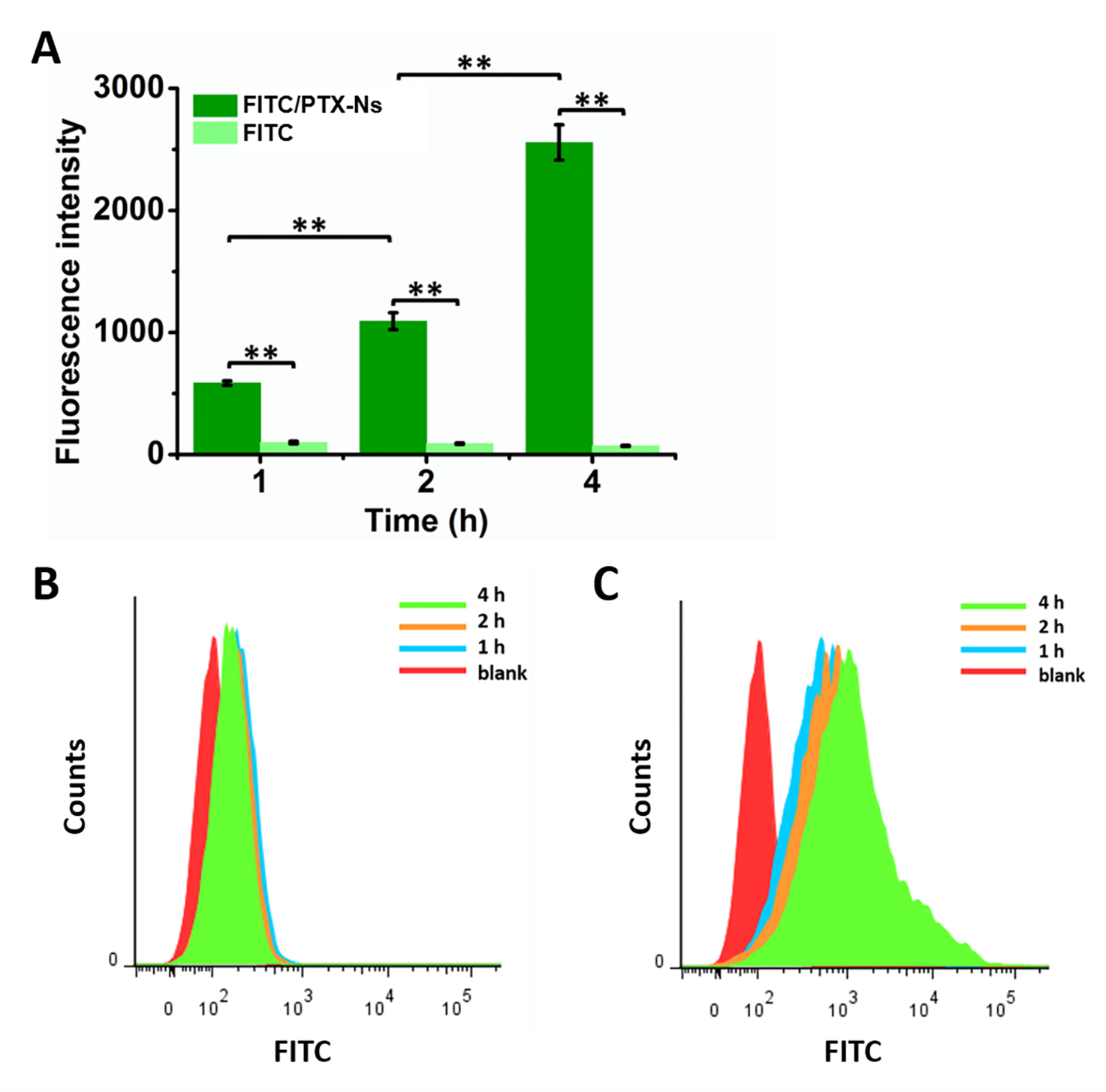


**Figure S2. Cellular uptake of FITC/PTX-Ns in a time-dependent pattern.** (A) Fluorescence intensity quantification and (B, C) flow-cytometry determination after incubation with FITC/PTX-Ns or free FITC (C, control) at a FITC concentration of 500 ng/mL at 37 °C for various durations. (mean ± S.D., *n* = 3, ***p* < 0.01).


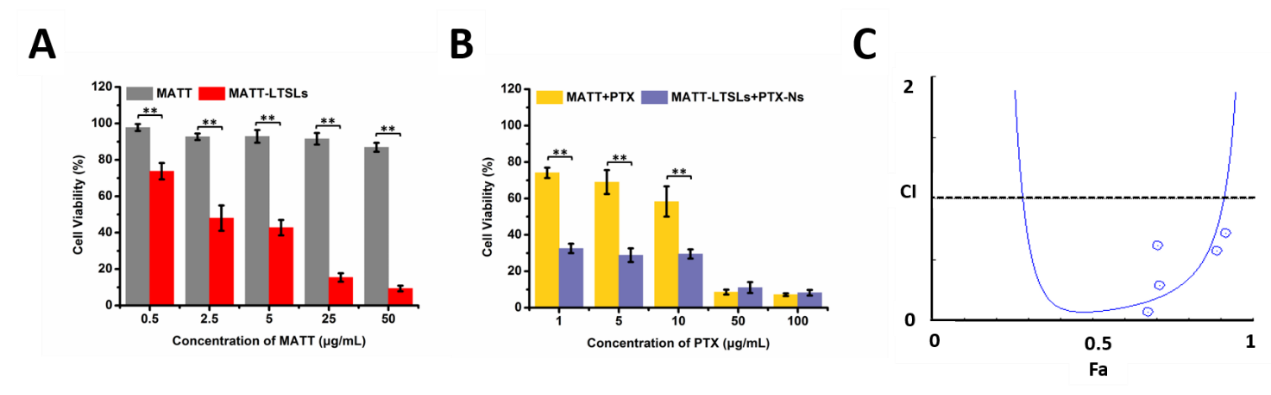


**Figure S3.** **Combination Index (CI) Calculation**. Cell cytotoxicity of 4T1 treated with (A) MATT formulations and (B) combined formulations of free drugs and nanomedicine for 48 h at 37 ^o^C (mean ± S.D., *n* = 5, ***p* < 0.01). (C) The curve of coefficient of drug interaction between MATT-LTSLs and PTX-Ns. *CI* was calculated by CompuSyn software. *CI* >1, *CI* <1, *CI* =1 indicate antagonistic effect, synergistic effect and addictive effect, respectively. The two nanomedicines showed the synergistic effect when the inhibition rate is between 0.3 and 0.9.


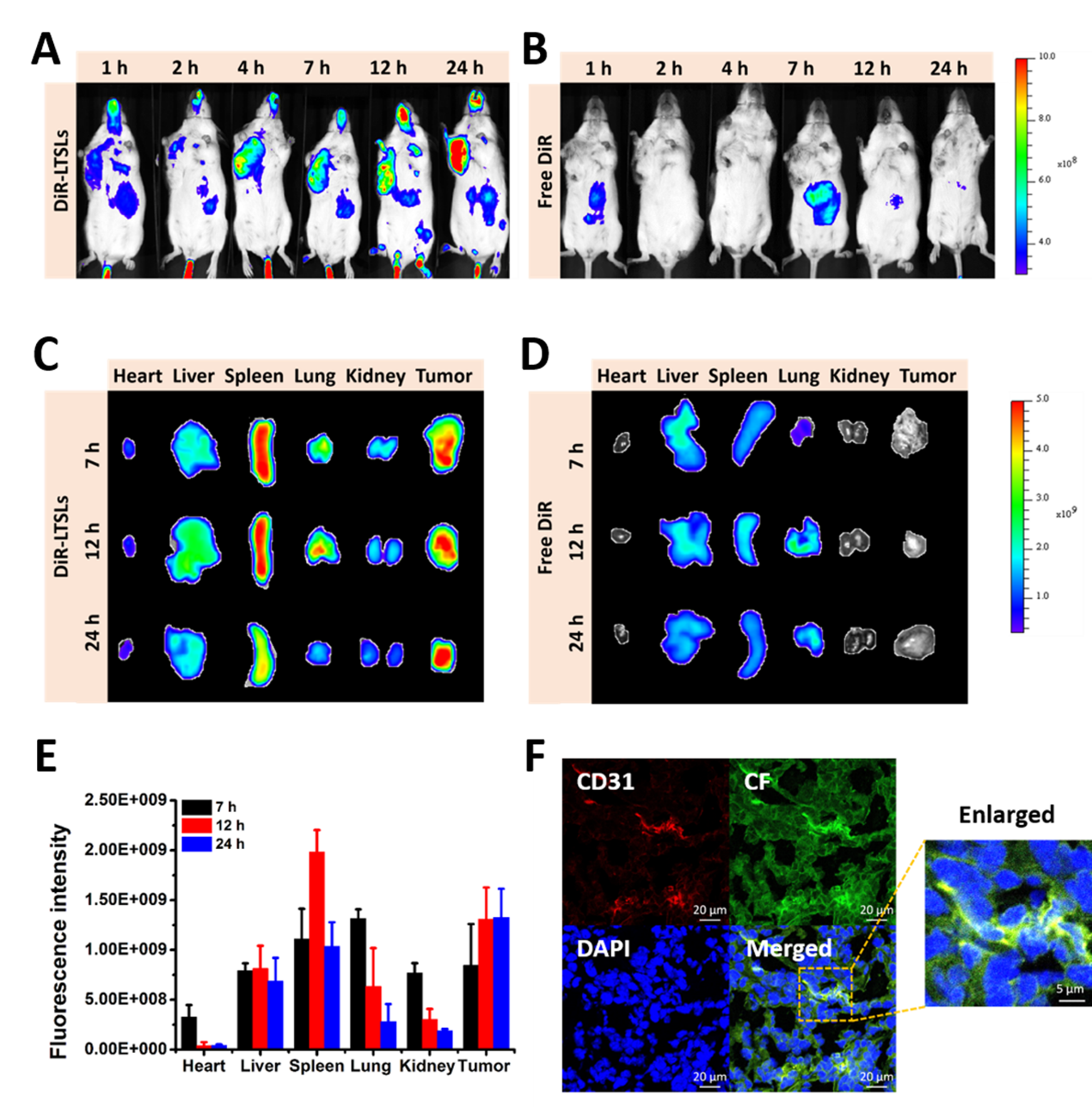


**Figure S4. Biodistribution of DiR-labeled LTSLs in 4T1 tumor-bearing Balb/C mice.** (A, B) Image of whole body at different time points after administration of (A) DiR-LTSLs and (B) free DiR through tail vein at the DiR dose of 0.5 mg/kg (*n* = 3). (C, D) *Ex* vivo image of major tissues collected at 7 h, 12 h, 24 h post injection of (C) DiR-LTSLs and (D) free DiR. (E) Fluorescence quantification of different tissues (mean ± S.D., *n* = 3). (F) The colocalization of CF-LTSLs (green) with microvessels stained with Cy7-labeled CD31 antibody (Red). The nucleus were stained with DAPI (blue). The yellow fluorescence spots indicated the colocalization. Scale bar of the enlarged view, 5 μm.


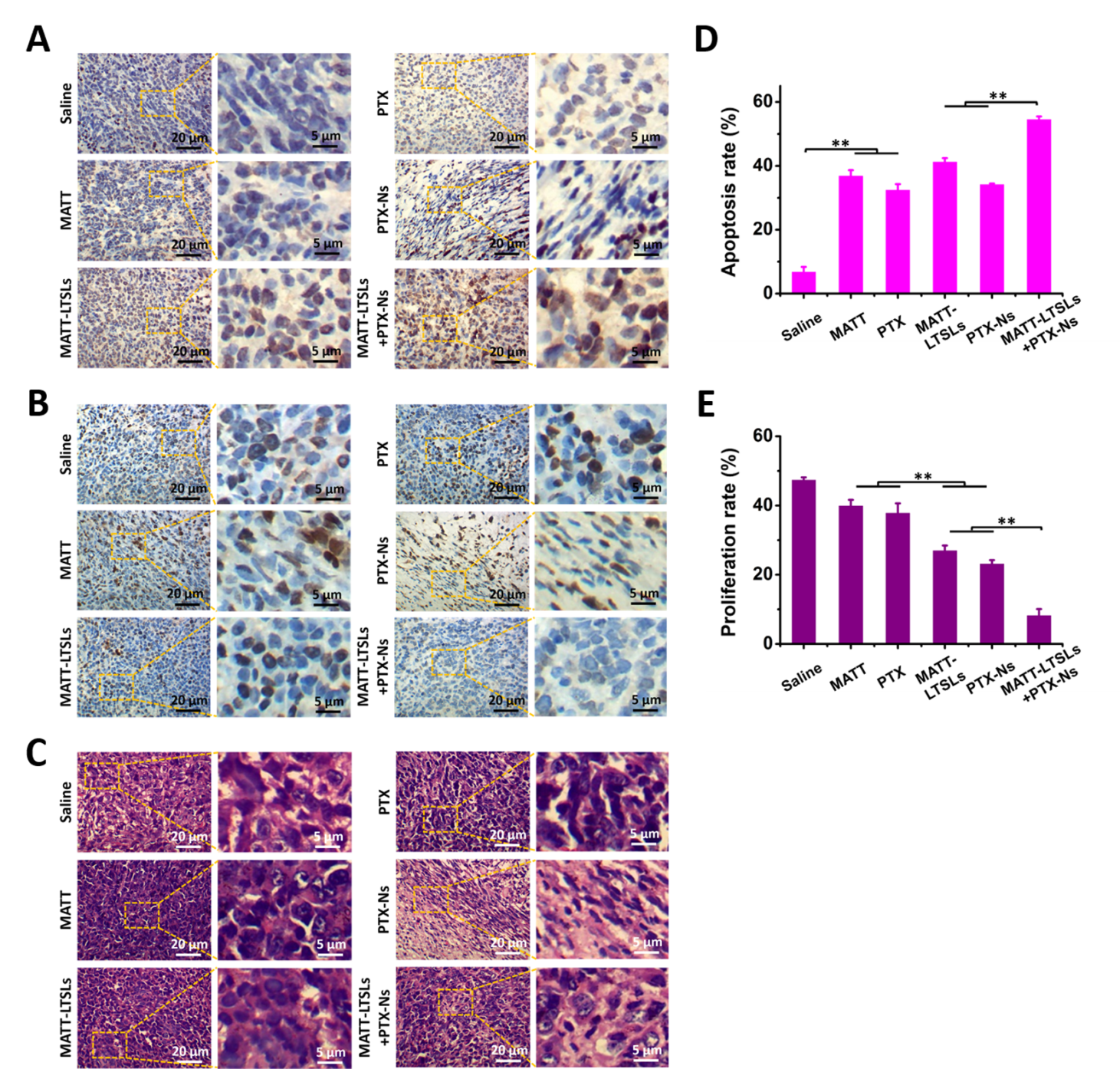


**Figure S5.** **H****istological study.** (A) TUNEL, (B) Ki67 and (C) H&E staining of sectioned tumor collected from 4T1 tumor-bearing Balb/C mice on day 16 after treatment. Quantitative analysis of tumor cell (D) apoptosis rate and (E) proliferation rate. Cell apoptosis and proliferation rate were quantified by five representative fields of cell nuclei under an optical microscope (mean ± S.D., *n* = 5, ***p* < 0.01). In H&E analysis, nuclei are stained blue, while extracellular matrix and cytoplasm are stained red.
